# Patterns of prescription drug expenditures and medication adherence among medicare part D beneficiaries with and without the low-income supplement

**DOI:** 10.1186/s12913-014-0665-3

**Published:** 2014-12-20

**Authors:** Stella M Yala, Obidiugwu Kenrik Duru, Susan L Ettner, Norman Turk, Carol M Mangione, Arleen F Brown

**Affiliations:** David Geffen School of Medicine at University of California, Los Angeles and Department of Medicine, Division of Cardiology, University of California, Los Angeles 10833 Le Conte Avenue A2-237 Center for the Health Sciences, Los Angeles, CA 90024 USA; David Geffen School of Medicine at University of California, Los Angeles, Department of Medicine, Division of General Internal Medicine & Health Services Research, 10940 Wilshire Boulevard, Suite 700, Los Angeles, CA 90024 USA; Jonathan Fielding School of Public Health, University of California, Los Angeles 911 Broxton Plaza, Los Angeles, CA 90024 USA

**Keywords:** Medicare Part D, Low-income subsidy, Gap coverage, Health care expenditures, Adherence to medications

## Abstract

**Background:**

The association between the Medicare Part D low-income subsidy (LIS), gap coverage, and outcomes such as medical expenditures, prescription fills, and medication adherence is not well understood. The purpose of this study was to examine the relationship between the LIS and these measures for patients within a large, national Part D plan in the United States.

**Methods:**

In this cross-sectional, retrospective analysis, we compared total and plan expenditures, out-of-pocket costs, and medication fills and adherence for three categories of Medicare beneficiaries: non-LIS beneficiaries without gap coverage (non-LIS/non-GC), non-LIS beneficiaries with gap coverage (non-LIS/GC), and LIS beneficiaries (LIS).

**Results:**

LIS beneficiaries, relative to non-LIS/non-GC and non-LIS/GC beneficiaries, had higher total expenditures ($1,887 vs. $1,360 vs. $1,341); lower out-of-pocket costs ($148 vs. $546 vs. $570); more expenditures exceeding the gap threshold (27.6% vs. 18.4% vs. 16.9%); and slightly higher adherence to blood pressure (65.6% vs. 64.2% vs. 62.4%); diabetes (62.5% vs. 57.7 vs. 57.4%); and lipid-lowering (59.6% vs. 57.0 vs. 55.6%) medications.

**Conclusion:**

LIS beneficiaries had higher total expenditures, lower out-of-pocket costs, and modestly better adherence to diabetes medications than non-LIS/non-GC and non-LIS/GC beneficiaries.

## Background

The centerpiece of the United States Medicare Prescription Drug, Improvement, and Modernization Act of 2003 was Medicare Part D, a subsidized pharmaceutical benefit that went into effect in 2006. This additional coverage—which provides outpatient prescription drug insurance to seniors and to people under age 65 with certain disabilities—constituted the most substantial expansion of the Medicare program since its inception in 1965. Under Medicare Part D, all enrollees receive a subsidy for prescription drug insurance; an additional low-income subsidy (LIS) is available to enrollees with sufficiently low income and assets [1-4]. Eligibility for the LIS and the generosity of the subsidy depend on the beneficiary’s income and assets. The LIS benefit applies to “dual eligible” beneficiaries who are enrolled in Medicare and Medicaid, but also covers those with incomes between 135 percent and 150 percent of the federal poverty level, who are not enrolled in Medicaid, and whose assets are below a given threshold. In 2011, about 10.5 million individuals, or 36% of Medicare Part D enrollees, received LIS [5].

By covering nearly all premiums for the basic benefit and by subsidizing most of the out-of-pocket costs, the United States government pays for approximately 95% of the spending for LIS beneficiaries, [5,6] who account for 75% of federal spending on Medicare Part D, 27% of all Medicare spending, and 39% of Medicaid spending [6]. The LIS benefit provides full or partial waivers for many out-of-pocket cost-sharing requirements, including premiums, deductibles, and medication co-payments. It also provides 100% of the cost of medications during the “coverage gap” (the difference between the initial coverage limit and the catastrophic coverage threshold). The out-of-pocket cost share of spending for LIS beneficiaries is considerably lower than that for non-LIS beneficiaries. The substantial differences in cost sharing, with otherwise identical benefit provisions, present an opportunity to assess how well the LIS program has achieved its goal of improved access to medications for low-income beneficiaries. Although Medicare Part D helps older adults realize savings, [7,8] costs related to non-adherence to pharmacotherapy remain a barrier, especially for low-income beneficiaries, and are associated with higher rates of hospitalization, emergency department use, and mortality [9-11]. Few previous studies have assessed the impact of LIS status and gap coverage on expenditures and adherence.

We examined total and out-of-pocket expenditures, the probability of having expenditures that exceeded the gap threshold, and the probability of adherence to medications among three groups of Medicare Part D beneficiaries: 1) LIS beneficiaries, 2) those without the LIS who had gap coverage for medications (non-LIS/GC), and 3) those without the LIS who did not have gap coverage (non-LIS/non-GC). Lower cost sharing among LIS beneficiaries was expected to lead to higher rates of medication and service use, resulting in increased total expenditures. Thus, we hypothesized that, compared to other Medicare Part D beneficiaries, after adjusting for other differences, LIS beneficiaries would have higher total expenditures, lower out-of-pocket costs, a higher probability of having expenditures that exceed the gap threshold, and a higher probability of adherence to medications used to treat chronic conditions, specifically, hypertension, diabetes, and hypercholesterolemia. We further hypothesized that Non-LIS/GC beneficiaries would fill more, or more expensive, prescriptions than non-LIS/non-GC beneficiaries.

## Methods

### Study design and population

**Figure 1:**
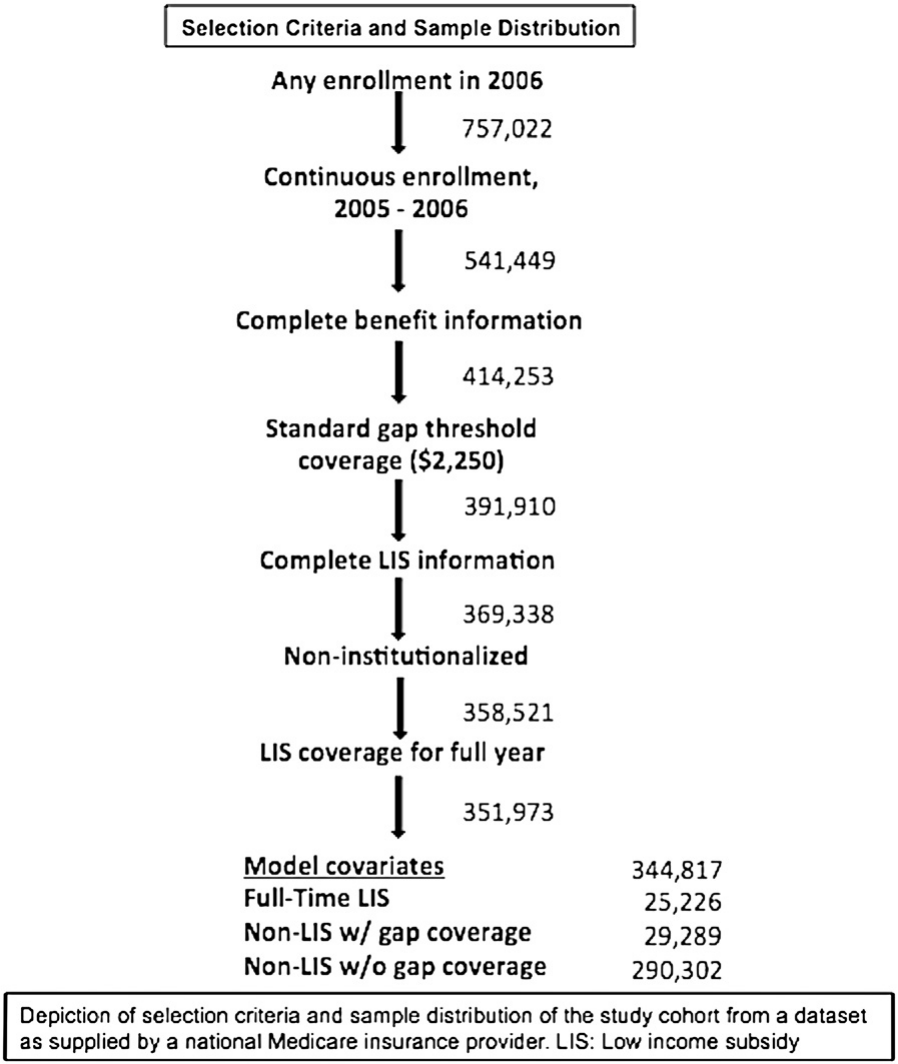
**Selection criteria and sample distribution.**

In these cross-sectional, retrospective analyses, we linked 2005–2006 data from Medicare Part D beneficiaries enrolled in a large national health care insurance carrier to 2000 United States Census data. Health plan data included enrollment files, pharmacy claims, and medical claims for Medicare Part D beneficiaries located in eight states within the country (Arizona, California, Colorado, Nevada, Oklahoma, Oregon, Texas, and Washington). To ensure complete capture of pharmacy data and prior-year diagnoses from the medical claims, participants had to be continuously enrolled from January 1, 2005 through December 31, 2006. As shown in Figure 1, the following groups were excluded from the analysis: 1) Patients whose insurance product or census tract could not be identified; 2) patients who had missing benefits or other information needed to categorize them as low-income beneficiaries; 3) patients with an extended gap threshold ($3,000); and 4) LIS beneficiaries who were institutionalized or who had intermittent LIS coverage (less than one full year). While CMS reporting typically classifies individuals as LIS recipients even if they qualified for only part of a given year, we elected to exclude this group as their copayments were likely to vary substantially during the study window. The Institutional Review Board of the University of California, Los Angeles, approved the study design, IRB#10-001460-CR-00004.

### Measures

The main predictor variable was LIS status, categorized as non-LIS/non-GC (the reference group); non-LIS/GC; and LIS beneficiaries, who automatically have gap coverage. Dependent variables, measured in 2006, included the following: 1) prescription drug expenditures including out-of-pocket, plan, and total medication expenditures; 2) whether expenditures exceeded (or in the case of those with gap coverage, would have exceeded) the 2006 gap threshold of $2,250; 3) number of prescription medications; and 4) adherence to medications for hypertension, diabetes, and hypercholesterolemia.

Out-of-pocket expenditures were comprised of patient costs of copayments, coinsurance, and deductibles. Plan expenditures were defined as all prescription costs that were not the patient’s burden. For LIS beneficiaries this included all costs attributable to the plan plus all government subsidies, whereas for non-subsidized beneficiaries this included only the cost burden to the plan. Total expenditures for medications were calculated as the sum of costs to the plan, out-of-pocket costs, and, for LIS beneficiaries, any government subsidies. Expenditure outcome measures were created for all filled prescriptions and also separately for only brand-name prescription drug fills.

The total number of medications was measured from data provided by health plan pharmacies for 2006, which included medications obtained through retail pharmacies and by mail order. Two measures of medication usage were used: 1) Total number of generic plus branded prescriptions and 2) count of branded prescriptions alone. To measure annual and monthly adherence to oral drugs for diabetes, hypertension, and hypercholesterolemia, the proportion of days covered (PDC) was calculated from dispensing data within each class of drugs. Adherence was defined as having a PDC ≥80% for the year, allowing drug supply to carry over from fill to fill. Each model of medication adherence included only beneficiaries with the condition of interest.

Covariates included demographic characteristics (age, gender) and co-morbid conditions (hypertension, hypercholesterolemia, coronary artery disease, presence of a mental health condition including depression and anxiety, dementia, osteoarthritis, rheumatoid arthritis, non-skin cancer, chronic obstructive pulmonary disease, congestive heart failure, atrial fibrillation/cardiac dysrhythmia, end-stage renal disease, stroke, peripheral vascular disease, and diabetes). Co-morbid conditions were identified using ICD-9-CM diagnosis codes from 2005 medical claims data and were classified into categories based on the Clinical Classifications Software [12]. As proxies for individual socioeconomic status, United States Census data were used to establish characteristics of the census tract where beneficiaries resided. The variables included median household income, percentage of population with less than a high school education, percentage with less than a college education, percentage by race/ethnicity, and percentage of linguistic isolation. The state of residence (Arizona, California, Colorado, Nevada, Oklahoma, Oregon, Texas, or Washington) was also included in models.

### Statistical analyses

Generalized linear models were constructed to assess the association between LIS status (LIS, non-LIS/GC, and non-LIS/non-GC [reference group]) and dependent variables including a) expenditures (total, out-of-pocket, and plan expenditures), b) number of prescriptions and supply of medications, and c) proportion of days covered (PDC) of diabetes, anti-hypertensive, and lipid lowering prescriptions. Logistic regression models were used to assess the association between LIS status and a) adherence to diabetes, anti-hypertensive, and lipid lowering medications (which was defined as a PDC of ≥80%), and b) reaching the 2006 Medicare Part D coverage gap expenditure threshold of $2,250. All models controlled for state of residence and the patient characteristics described above. Analyses were conducted using SAS version 9.3.

To minimize the effect of unmeasured group differences, propensity score analyses were performed as sensitivity tests of the robustness of the study findings. Multivariate analyses comparing the LIS, non-LIS/GC, and non-LIS/non-GC sample were repeated using propensity score techniques to create analytic groups that were better matched on observed individual and census variables. Stata v. 10.1 was used to construct propensity models that included the demographic, comorbidity, and census-level covariates from the main analyses to predict the likelihood of being a member of the LIS group. The sample was then divided into five parts, corresponding to quintiles of the propensity score distribution. Each of the outcomes was then re-estimated using the observations within each propensity score stratum.

## Results

### Characteristics of the study population

The final study sample comprised 344,817 beneficiaries, of whom 25,226 were LIS, 29,289 were non-LIS/GC, and 290,302 were non-LIS /non-GC (Table 1). Among the LIS beneficiaries, 74% were female as compared with 61% female among the non-LIS/GC group, and 60% female. In unadjusted results, several chronic conditions were more common in the LIS group than in the non-LIS/GC and the non-LIS/non-GC groups, respectively, including diabetes mellitus (31.6%, 23.8%, 24.5%); congestive heart failure (17.8%, 14.0%, 12.5%); peripheral vascular disease (15.3%, 11.2%, 12.8%); and dementia (12.0%, 7.4%, 6.8%), among others (Table 1).

**Table 1:** **Characteristics of the study population and unadjusted outcomes stratified by type of medicare part D coverage (n = 344,817)**

	**LIS N = 25,226**	**Non-LIS/GC N = 29,289**	**Non-LIS/Non-GC N = 290,302**
**Age, years (SD)**	78.8 (7.2)^a,b^	78.6 (7.0)^a^	77.7 (6.7)
**Gender (% female)**	73.9^a,b^	60.8^a^	59.6
**Co-morbid conditions**			
Hypertension (%)	77.0^a,b^	73.0^a^	74.1
Hyperlipidemia (%)	52.4^a^	52.4^a^	58.3
Osteoarthritis (%)	33.1^a^	32.4^a^	31.5
Diabetes (%)	31.6^a,b^	23.8^a^	24.5
Chronic Obstructive Pulmonary Disease (%)	27.9^a,b^	20.1^a^	23.2
Coronary Artery Disease (%)	26.9^a,b^	23.3^a^	25.0
Atrial fibrillation (%)	25.8^a^	25.2^a^	24.7
Non-skin cancer (%)	23.6^a,b^	33.9^a^	31.3
Stroke (%)	20.7^a,b^	17.5^a^	17.0
Mental health (%)	18.0^a,b^	15.9^a^	13.8
Congestive Heart Failure (%)	17.8^a,b^	14.0^a^	12.5
Peripheral Vascular Disease (%)	15.3^a,b^	11.2^a^	12.8
Dementia (%)	12.0^a,b^	7.4^a^	6.8
End-stage renal disease (%)	5.9^a,b^	4.6^a^	4.9
Rheumatoid arthritis (%)	3.5^a,b^	2.4^a^	2.8
**Expenditures 2006**			
Total costs (SD)	$2,085 (2,411)^a,b^	$1,290 (1,495)	$1,275 (1,439)
Total out-of-pocket costs (SD)	$159 (224)^a,b^	$511 (608)^a^	$544 (662)
Plan costs (SD)^c^	$1,926 (2,316)^a,b^	778 (1,028)^a^	$731 (951)
Total cost of brand name drugs (SD)	$1,359 (2,105)^a,b^	$729 (1,263)^a^	$766 (524)
Out-of-pocket cost of brand name drugs (SD)	$93 (178)^a,b^	$301 (533)^a^	$318 (524)
Plan cost of brand name drugs (SD)^c^	$1,266 (2,034)^a,b^	$428 (868)^a^	$448 (857)
**Total number of prescriptions (SD)**	42 (33)^a,b^	25 (21)^a^	26 (22)
**Total number of BM (SD)**	12 (14)^a,b^	6 (8)^a^	7 (9)
**Medication adherence**			
Diabetes medications, PDC (%)	76.8	77.3^a^	76.1
Diabetes medications, % adherence	59.4^a^	58.9	57.7
Hypertension medications, PDC (%)	79.3^a,b^	80.1^a^	78.6
Hypertension medications, % adherence	63.7^a,b^	65.7^a^	62.5
Lipid-lowering medications, PDC (%)	73.2^a,b^	75.8^a^	73.0
Lipid-lowering medications, % adherence	56.9^a,b^	59.8^a^	55.6

LIS: Low-income subsidy beneficiaries; GC: gap coverage; SD: standard deviation; PDC: the proportion of days covered, which was calculated from dispensing data within each class of drugs; Adherence: defined as having a PDC ≥80% in the year or month for the entire regimen, allowing drug supply to carry over from month to month. Each model of medication adherence included only beneficiaries with the condition of interest.

^a^Significantly different from the Non-LIS/Non-GC group (p <0.05).

^b^Significantly different from the Non-LIS/GC group (p <0.05).

^c^Measures of plan cost for LIS beneficiaries is comprised of the cost to the plan plus subsidies.

There were significant differences by LIS status in census-based measures of race/ethnicity, median household income, and education (Table 2). Among LIS beneficiaries, there was a higher proportion of Latinos relative to whites or African Americans, and more linguistic isolation among the residents of their census tract compared to the other two groups. The median household income for LIS beneficiaries was lower, at $42,412 (SD $19,206), than non-LIS/GC and non-LIS/non-GC beneficiaries, whose median household incomes were $51,022 (SD $19,878) and $51,459 (SD $22,397), respectively. Finally, on average LIS beneficiaries resided in zip codes where 25% of residents with ages ≥25 years had not completed high school, compared to 18% for non-LIS/non-GC beneficiaries and 16% of non-LIS/GC beneficiaries.

**Table 2:** **Characteristics of patients in residential census tracts stratified by type of medicare part D coverage (n = 344,817)**

	**LIS N = 25,226**	**Non-LIS/GC N = 29,289**	**Non-LIS/Non-GC N = 290,302**
**Median household income (SD)**	$42,412 (19,206)^a,b^	$51,022 (19,878)^a^	$51,459 (22,397)
**Education level (%)**			
Percentage of residents with < high school education	25.1^a,b^	16.4^a^	17.8
Percentage of residents with < college degree	74.3^a,b^	65.3^a^	66.8
Proportion of residents with linguistic isolation	8.1^a,b^	5.3^a^	5.5
**Race/Ethnicity (%)**			
White	72.6^a,b^	80.7^a^	80.3
Latino	29.4^a,b^	20.2^a^	19.8
Other race	15.4^a,b^	10.5^a^	9.9
African American	7.9^a,b^	3.9^a^	5.0
Asian/Pacific Islander	5.7^a,b^	6.9^a^	6.1
American Indian	2.4^a,b^	2.1^a^	2.0

SD: standard deviation.

^a^Significantly different from the Non-LIS/Non-GC group (p <0.05).

^b^Significantly different from the Non-LIS/GC group (p <0.05).

### Adjusted plan and prescription costs

Adjusted health plan and beneficiary out-of-pocket prescription drug expenditures are shown in Table 3. Compared to total drug expenditures in 2006 for the non-LIS/non-GC reference group ($1,341), total expenditures were higher for the non-LIS/GC group ($1,360, p < 0.05) and the LIS group ($1,887, p < 0.01). Similarly, out-of-pocket expenses were higher for the non-LIS/non-GC reference group ($570) than for the non-LIS/GC group ($546, p < 0.01) and for the LIS group ($148, p < 0.01). Compared to the reference group, total and plan expenditures on brand name medications were higher in the LIS and non-LIS/GC groups (p < 0.05 for all); however, out-of-pocket expenses on branded medications were lower for the LIS group (p < 0.01) and higher for the non-LIS/GC group (p < 0.05) relative to the reference group. The adjusted percentages of patients with expenditures that exceeded the gap threshold were 27.6% in the LIS group (p < 0.01) and 17.4% in the non-LIS/GC (p < 0.05) compared with 16.9% in the non-LIS/non-GC group.

**Table 3:** **Regression-adjusted estimates of expenditures, prescription drug use, and adherence to medications stratified by type of medicare part D coverage (n = 344,817)**

	**LIS N = 25,226**	**Non-LIS/GC N = 29,289**	**Non-LIS/Non-GC N = 290,302**
**Expenditures**			
Total expenditures	$1,887^a,b^	$1,360^a^	$1,341
	(1,864-1,910)	(1,344-1,375)	(1,336-1,347)
Out-of-pocket expenditures	$148^a,b^	$546^a^	$570
	(146–150)	(539–552)	(567–572)
Plan expenditures^c^	$1,708^a,b^	$822^a^	$776
	(1,687-1,729)	(811–833)	(772–780)
**Expenditures on brand name medications**			
Total expenditures	$1,325^a,b^	$926^a^	$898
	(1,305-1,346)	(911–941)	(893–903)
Out-of-pocket expenditures	$96^a,b^	$374^a^	$369
	(94–98)	(368–381)	(367–371)
Plan expenditures^c^	$1,221^a,b^	$560^a^	$537
	(1,202-1,240)	(549–571)	(533–540)
**Expenditures exceeding the gap threshold**	27.6%^a,b^	17.4%^a^	16.9%
	(27.2-28.1)	(17.0-17.8)	(16.8-17.0)
**Prescriptions**			
Total number of prescriptions	38.1^a,b^	25.1^a^	26.5
	(37.8-38.4)	(24.9-25.3)	(26.4-26.5)
Total number of brand name prescriptions	10.7^a,b^	6.4^a^	6.9
	(10.5-10.8)	(6.3-6.5)	(6.87-6.92)
**Adherence to medications**			
Diabetes drug adherence	62.5%^a,b^	57.7%	57.4%
	(61.0-63.9)	(56.0-59.4)	(56.9-58.0)
Hypertension drug adherence	65.6%^a,b^	64.2%^a^	62.4%
	(64.9-66.3)	(63.5-64.9)	(62.2-62.6)
Lipid-lowering drug adherence	59.6%^a,b^	57.0%^a^	55.6%
	(58.5-60.6)	(56.0-58.0)	(55.3-55.9)

LIS: low-income subsidy beneficiaries; GC: gap coverage; PDC, proportion of days covered, which was calculated from dispensing data within each class of drugs; Adherence: defined as having a PDC ≥80% in the year or month for the entire regimen, allowing drug supply to carry over from month to month.

Each model of medication adherence included only beneficiaries with the condition of interest. Estimates were adjusted for the individual beneficiary characteristics and clinical co-morbidities listed in Table 1 and for the residential census characteristics listed in Table 2.

^a^Significantly different from the Non-LIS/Non-GC group (p <0.05).

^b^Significantly different from the Non-LIS/GC group (p <0.05).

^c^Measures of plan cost for LIS beneficiaries is comprised of the cost to the plan plus subsidies.

### Prescription fills and adherence to medications

Compared to the non-LIS/non-GC group, which filled an average of 26.5 prescriptions, including 6.9 branded medication prescriptions during 2006, LIS participants filled more prescriptions (38.1 total, 10.7 branded; p < 0.01), and the non-LIS/GC group filled fewer prescriptions (25.1 total, 6.4 branded; p < 0.01) (Table 3).

Relative to the non-LIS/non-GC group, participants in the LIS group were slightly more likely to be adherent to medications for diabetes (62.5% vs. 57.4%), and to a lesser degree hypertension (65.6% vs. 62.4%), and hypercholesterolemia (59.6% vs. 55.6%, p < 0.01 for all) (Table 3). Relative to the reference group, non-LIS/GC participants had slightly higher rates of adherence to medications for hypertension (64.2% vs. 62.4%) and hypercholesterolemia (57.0% vs 55.6%; p < 0.01 for both), but not to medications for diabetes.

### Sensitivity analyses

Results of propensity analyses for all expenditure, prescription/supply, and gap threshold models for all five sub-samples were consistent with the reported results. Results for adherence were also largely consistent in direction with the results above, but several results did not reach statistical significance. The difference between the main analysis and the propensity analyses appears to be due to the relatively small LIS sample within each stratum, as not every subject was on a condition-specific medication. For example, only 94 subjects within the lowest propensity score quintile were prescribed a diabetes medication.

## Discussion

The effects of the LIS on expenditures (total, out-of-pocket, and plan) and its association with medication adherence among Medicare Part D beneficiaries in the United States have not previously been adequately explored. The few published articles on outcomes related to LIS have focused on the transition from Medicaid to Medicare for those who are dual-eligible (Medicare beneficiaries who qualify for full Medicaid benefits) [13-15] and have compared subsidized with un-subsidized diabetic beneficiaries enrolled in the same prescription drug plans [11]. In the present study, consistent with our hypothesis, LIS beneficiaries had higher total expenditures, lower out-of-pocket costs, and were more likely to reach the gap expenditure threshold relative to non-LIS beneficiaries. They had more filled prescriptions and slightly higher adherence primarily to medications for diabetes. Among non-LIS Medicare Part D beneficiaries, those who had coverage in the gap had higher total expenditures, and modestly lower out-of-pocket costs, but did not have consistently higher medication refills or adherence relative to non-LIS/non-GC beneficiaries.

Several factors may contribute to higher spending among LIS beneficiaries. Among all beneficiaries, higher spending is driven by those who fill more (and more expensive) prescriptions [5]. The complex and intensive medical needs of the LIS beneficiaries in this study may have resulted in the higher total expenditures, and with the current study design it is difficult to definitively establish whether these higher expenditures were due to medical complexity or a richer benefit. The present results show that LIS beneficiaries have more comorbid conditions *and* use more health care services and prescription medications than either category of non-LIS beneficiaries.

On average, the LIS group filled more prescriptions than those in the non-LIS/non-GC group, even after adjusting for chronic conditions. The higher spending may also reflect more generous coverage through the LIS benefit. The LIS resulted in substantially lower out-of-pocket expenditures relative to the non-LIS/GC and the non-LIS/non-GC groups, a factor that may have contributed to the higher rates of service use and total expenditures observed among LIS beneficiaries.

Once the out-of-pocket threshold is reached, the beneficiary becomes eligible for catastrophic coverage. During catastrophic coverage, the beneficiary pays a percentage of coinsurance, or a specific amount of generic drugs and brand-name drugs. The adjusted rate of expenditures that exceed the gap threshold was greater for the LIS group relative to non-LIS/non-GC group. This finding may be attributed to the fact that older individuals with chronic diseases requiring pharmaceutical therapy are more likely to have expenditures that exceed the gap threshold. The cost sharing provided by the LIS limits their out-of-pocket spending and effectively eliminates the coverage gap. LIS enrollees, for whom the gap is eliminated, account for more than half of the enrollees with spending high enough to reach the expenditures that exceed the gap threshold [16].

Our study underscores the importance of pharmaceutical drug subsidies to help low-income patients in the United States self-manage their chronic conditions. Data from the 2010 Organization for Economic Cooperation and Development (OECD) survey shows that annual per capita pharmaceutical spending in the United States ($897) is significantly higher than other industrialized countries [17]. Per capita pharmaceutical spending is lower in the United Kingdom ($368), France ($607) and Germany ($563), but each of these nations still has a more generous health care and pharmaceutical subsidy in place for low-income patients as compared to the United States [17]. Specifically, the United Kingdom has little or no cost-sharing for any medical spending, France waives cost-sharing for people with any of 30 chronic conditions (including diabetes), and Germany caps out-of-pocket health expenses at 1-2% of income [18]. The up-front societal expense of providing these subsidies is likely to translate to improved health outcomes across the population. As might be expected, rates of “preventable” mortality by country, defined as mortality from treatable conditions such as diabetes, is lower in each of these 3 countries as compared with the United States [19]. Taken in an international context, the relatively modest LIS pharmaceutical subsidy for low-income Medicare recipients may be contributing to improved health outcomes, and additional studies should examine the potential additional benefits of increasing number of enrollees and the generosity of the subsidy.

An important clinical consequence of the higher rate of spending and of medication fills among LIS beneficiaries may be better adherence to medications to treat common chronic conditions such as diabetes, hypertension, and hypercholesterolemia, which in turn has been associated with reduced cardiovascular risk [20] and lower medical costs [21]. We found evidence of modestly higher adherence to medications primarily for diabetes among LIS beneficiaries relative to non-LIS/non-GC beneficiaries but less pronounced differences between those in the non-LIS/GC and non-LIS/non-GC categories. Even after implementation of Medicare Part D, the sickest segments of the Medicare population have persistently high levels of cost-related medication non-adherence and spend less on basic needs in order to be able to afford medications [22,23]. More research is needed to characterize the relationship of costs to other factors that may influence adherence, including adequate clinical follow-up, patients’ perception of the benefits of treatment, provider-patient relationships, co-morbid conditions (especially mental health problems), and polypharmacy [23]. This pattern of increased adherence for LIS beneficiaries is not seen across all studies, as a recent analysis of a large Medicare cohort found that these individuals had lower adherence than a non-LIS comparison group, although this analysis did not control for comorbid conditions [24]. Additionally, the impact of the modestly increased adherence observed in the present study on long-term clinical outcomes is not well understood, and the financial burden associated with higher costs among non-LIS beneficiaries and whether they lead to unacceptable tradeoffs for this group is unknown.

The present study has potential limitations. First, since the data were drawn from only eight, predominantly western, states and institutionalized beneficiaries were excluded; the findings may not be nationally generalizable. Nonetheless, most Medicare Part D enrollees are in for-profit plans, and the for-profit Medicare Part D plan evaluated was among the largest in the country. Another concern is that the LIS definition is based on evidence of low income during a calendar year, but some patients may have belonged to the LIS category for only part of the year; however, such misclassification is likely to have resulted in an underestimation of the differences between the LIS and non-LIS categories and we have excluded those whose LIS coverage was less than one year. Additionally, while they provide insight into real world patterns, claims data are collected for the purpose of payment, not research, and may not accurately capture all relevant aspects of a patient's medical history. Thus, although claims provide insight into prescription medication use, the presence of a prescription claim does not guarantee that patients took medications as prescribed. We relied on census tract data as proxies for individual characteristics such as income, education, and race. Although this approach has been used extensively in analysis of administrative data, the direction and extent of misclassification introduced is difficult to estimate. LIS status may be a proxy for other characteristics such as low health literacy, which in turn may be linked to underlying risk factors and health-related behaviors and not to the specific medication subsidy that LIS patients receive. Finally, the mix of available branded and generic medications has changed since this data was collected, and some of the results related to costs and classes of medications used may not be representative of current utilization patterns.

## Conclusion

Our findings suggest that gap coverage may be associated with improved medication adherence for LIS participants, primarily adherence to diabetes medications. Attempts to reduce or eliminate gap coverage may result in lowered adherence among the most vulnerable beneficiaries, leading to increased costs, poorer adherence and worse health outcomes. Further research will be needed to elucidate how extension of benefits under the Affordable Care Act will influence expenditures and adherence among Medicare beneficiaries in the United States who are not currently eligible for the subsidy.
